# Early life stress and hippocampal neurogenesis in the neonate: sexual dimorphism, long term consequences and possible mediators

**DOI:** 10.3389/fnmol.2015.00003

**Published:** 2015-02-13

**Authors:** Naima Lajud, Luz Torner

**Affiliations:** División de Neurociencias, Centro de Investigación Biomédica de Michoacán, Instituto Mexicano del Seguro SocialMorelia, Mexico

**Keywords:** hippocampus, stress, neuroplasticity, stress mediators, gender differences

## Abstract

Adverse early life experience decreases adult hippocampal neurogenesis and results in increased vulnerability to neuropsychiatric disorders. Despite that the effects of postnatal stress on neurogenesis have been widely studied in adult individuals, few efforts have been done to evaluate its immediate effects on the developing hippocampus. Moreover, it is not clear whether postnatal stress causes a differential impact in hippocampus development in male and female neonates that could be related to emotional deficits in adulthood. It has been proposed that the long term effects of early stress exposure rise from a persistent HPA axis activation during sensitive time windows; nevertheless the exact mechanisms and mediators remain unknown. Here, we summarize the immediate and late effects of early life stress on hippocampal neurogenesis in male and female rat pups, compare its later consequences in emotionality, and highlight some relevant mediator peptides that could be potentially involved in programming.

## INTRODUCTION

Early life stress influences behavioral and physiological functions in the individuals, and results in long term changes in neuronal function increasing the vulnerability to suffer a psychiatric diseases (Felitti et al., 1998; Heim and Nemeroff, 2001). The hippocampus is involved in cognitive functions, and is an important regulator of emotional responses to stress, as it is one of the brain structures involved in glucocorticoid (GC) mediated HPA axis negative feedback. It is also one of the two niches where new neurons are actively produced throughout life. Early exposure to adverse experiences induces permanent changes in brain development that include alterations of the hippocampal formation and a reduced or altered neural plasticity. Since the hippocampus is especially vulnerable to GC induced toxicity, it has been hypothesized that stress exposure during sensitive time windows causes alterations on hippocampal development leading to a vicious circle, which perpetuates and exacerbates the long term consequences of early life stress (Sapolsky and Meaney, 1986). However, it is not totally clarified whether changes in neurogenesis originate as a result of many alterations in the hippocampal structure along the time line, or are an immediate consequence of stress exposure. Also, it is unclear whether neural plasticity is equally affected in male and female subjects at early ages. In the present work we will discuss the effects of early life stress on hippocampal developmental neurogenesis and will include a short summary of some possible mediators of stressful effects.

## THE STRESS RESPONSE

Stress involves the activation of the autonomic nervous and neuroendocrine systems to release a cascade of neurotransmitters, hormones, and other chemical messengers that induce behavioral and metabolic changes in the organism. A fast response is conveyed by the autonomic nervous system. A delayed response activates the hypothalamic paraventricular nucleus (PVN) to release corticotropin releasing hormone (CRH) to the portal vasculature of the anterior pituitary gland; CRH stimulates the release of adrenocorticotropin hormone (ACTH) which triggers the release of GCs from the adrenal cortex. GCs exert a negative feedback regulating HPA axis activity via its own receptors [glucocorticoid receptors (GRs) and mineralocorticoid receptors (MRs)] in anterior pituitary, hypothalamus (de Kloet et al., 2005a), hippocampus and prefrontal cortex. In adults, the effects of allostatic load dissipate following the removal of the stressor; however, the effects of early life stress are persistent far beyond the period of stress exposure.

## ANIMAL MODELS OF EARLY LIFE ADVERSITY

Animal models that reproduce many of the features of chronic stress or adverse experiences during early life include prenatal stress (PS) exposure (Lemaire et al., 2000), acute maternal deprivation (MD) procedures (de Kloet et al., 2005b), chronic or periodic maternal separation (MS) models (Sanchez et al., 2001; Huot et al., 2002; Plotsky et al., 2005), chronic early life stress (CES; Ivy et al., 2008), and early weaning of the pups (Kikusui and Mori, 2009). During late pregnancy, maternal GC mediate changes in fetal HPA responsiveness that is already functional. Infant rodents spend their first weeks of life in the maternal nest; hence, interactions of the pups with their mother and littermates are essential for optimal brain development and social skills (Sanchez et al., 2001; Huot et al., 2002). Separation from the dam for prolonged periods (>2 h) is perceived as a threat by the offspring, and activates the neonate’s HPA axis, causing elevated basal and/or stress-induced corticosterone levels in the adult (Lajud et al., 2012a). MD is an acute traumatic event that consists on separating the offspring from the dam for a 24 h period, and involves both nutritional and sensory (stimulation) factors (Suchecki et al., 1993), while MS is a chronic moderate stressor that involves daily separations from the dam during the first 2 or 3 weeks of life. It has been proposed that adult phenotype induced by MS is programmed by the pup’s stressful experience during prolonged MS, rather than by prolonged maternal absence *per se* (Daskalakis et al., 2014). Variations in the MS model include daily separations of 3 h (MS180) or more (MS360), once or twice a day, from days 1–14, 2–21, or 15–21, etc. (Nishi et al., 2014). The CESs model, interferes with the mother infant interaction through the induction of a reduced maternal care, due to poor housing conditions (scarce material to build a nest) from PN2 to PN9, and resembles maternal anxiety and neglect (Ivy et al., 2008). These models reproduce many of the consequences observed in humans subjected to adverse early experiences, such as infant maltreatment or abuse, low socio economic status, etc., (Sanchez et al., 2001; Huot et al., 2002; Plotsky et al., 2005), in terms of a chronic exposure to adverse situations. Since effects of PS on neurogenesis have been more studied, we will review the studies on early neurogenesis focusing more on the effects of postnatal stress.

## DEVELOPMENT OF THE HIPPOCAMPUS

**FIGURE 1:**
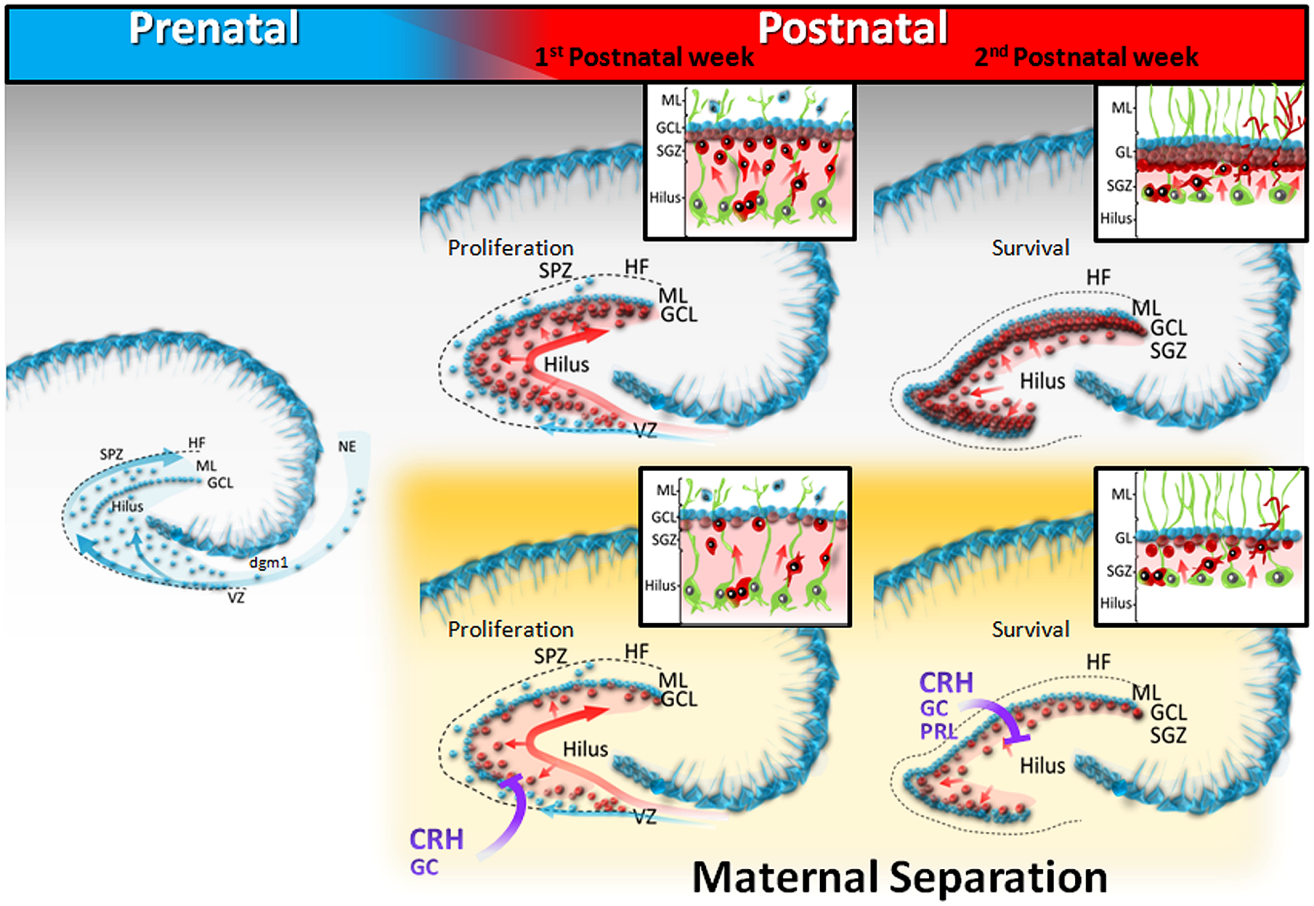
**Schematic diagram of dentate gyrus development in postnatally stressed pups.** During prenatal development (E17-22), the granule cells of the outer shell (blue) originate from the neuroepithelium (NE), and migrate to the subpial zone (SPZ), or traverse the hilus. Throughout the first postnatal week, the precursor cells build up a new proliferation zone distributed within the hilus (light red), and granule cells of the GCL inner core migrate, following the arrangement of the secondary radial glial scaffold (green). During the second week of life, the neurogenic niche is confined to the subgranular zone (SGZ). Maternal separation decreases both proliferation and survival of new neurons, generated in the hilar tertiary dentate matrix, probably through stress – mediated mechanisms.

The development of the rodent dentate gyrus (DG) can be subdivided into two major phases. First, the granule cells of the outer shell (**Figure 1**, blue) originate prenatally from the neuroepithelium (NE) located near the fimbria and migrate from the progressively receding secondary dentate matrix to the subpial zone (SPZ; **Figure 1**, blue). The first dentate migration (dgml) is the source of the earliest generated granule cells that will constitute the outer shell of the granular layer (Altman and Bayer, 1990a,b; Li et al., 2009). During the second postnatal phase (**Figure 1**, red), the precursor cells build up a new proliferation zone distributed within the hilus, and the early embryonic radial glial scaffold from the ventricular zone (VZ) is replaced by a secondary glial scaffold that traverses the hilus (**Figure 1**, green). Most radial glial cells, support migrating neurons and serve as precursor cells for both neurogenesis and gliogenesis (Brunne et al., 2013). This tertiary dentate matrix peaks its proliferation rate between PND3 and PND10 and is responsible for the great increase in granule cell population during the neonatal period (Bayer, 1980). The granule cells (**Figure 1**, red) colonize either the outer shell or the inner core of the granule cell layer (GCL) in a symmetrical manner (Martin et al., 2002), and neurogenesis follows a characteristic dorso – ventral maturation gradient (Schlessinger et al., 1975). During the third and fourth weeks of life, the tertiary dentate matrix disappears and henceforth the neurogenic niche becomes largely confined to the subgranular zone (SGZ; Altman and Bayer, 1990b). This SGZ is the main source of granule cells produced during early life and adulthood. For lifelong neurogenesis to occur, the DG must maintain the appropriate precursor cell niche in the SGZ, which is likely to be dependent on the developmental mechanisms at play during the DG formation. Stress exposure during the first weeks of life may have a significant impact on the maturation of the DG, as it disrupts the organization of the secondary and/or tertiary dentate matrix, altering permanently the structure and function of the hippocampus immediately after stress exposure.

## EARLY LIFE STRESS AND HIPPOCAMPAL NEUROGENESIS

Differences in neurogenesis between male and female pups have been recognized. More new BrdU+ cells were found in the DG of male rat pups compared to females at PN1 and PN4 (Zhang et al., 2008). Control males showed a higher proliferation rate, and increased survival of newborn cells, compared to control females. In addition, a larger granular cell layer volume and more young neurons (DCX) was found in males (Oomen et al., 2009). However, other group reported no differences on neurogenesis rates between male and female pups at PN15 (Lajud et al., 2013).

Early life adverse effects on adult hippocampal neurogenesis have been widely evaluated (Korosi et al., 2012), however the early effects during stress exposure period remain unclear. Immediate effects of stress exposure on hippocampal developmental neurogenesis, were initially addressed by Tanapat et al. (1998) who showed a reduced proliferation in the DG of male rat pups 24 h after a single stress exposure. MS also decreased the granular cell number in juvenile rats (Oreland et al., 2010). Early weaning in mice at PN15 induced fewer BrdU+ cells in the DG of male, but not female mice (Kikusui and Mori, 2009). MS (6 h/day) followed by early weaning (PN15) decreased cell proliferation in the DG of juvenile male rats (Baek et al., 2011, 2012). We showed that MS180 decreased the number and the density of BrdU+ cells in the DG of male pups, at PN15 (Lajud et al., 2012a). In contrast, increased cell proliferation and differentiation in the DG was found in male pups using the CES model (PND2 to PND9; Naninck et al., 2014). Since increased basal corticosterone levels were observed in CES pups (Naninck et al., 2014) but not after MS180 (Lajud et al., 2012a), we cannot exclude the possibility that the etiology of the adverse stimulus could exert differential effects. Maternally deprived pups (24 h PN3), showed decreased cell proliferation but not cell survival at PN21 (Oomen et al., 2009). In opposition to the studies in male pups, there are fewer reports concerning females. The number of BrdU+ cells was unchanged in the DG of female mice in response to early weaning (Kikusui and Mori, 2009). Naninck et al. (2014) showed an increased cell proliferation and differentiation in the DG of female CES pups. Preliminary studies from our group showed a decrease of cell survival in the DG of rat female pups at PN15, after MS180 (Lajud et al., 2012b). MD at PN3 found no changes in cell proliferation or survival in the DG of female pups at PN21, but only a decrease in the number of immature neurons (Oomen et al., 2009; **Table 1**).

**Table 1:** Immediate effects of early life stress on neurogenesis, and long term effects on emotionality, in male and female rodents.

Authors	Early life stress procedure	Species	Sex	Age	Effects on hippocampus	Emotional behavior in adulthood
Tanapat et al. (1998)	Acute exposure to the odor of a predator on PN6	Rats	Males	PN7	Decreased cell proliferation in the DG.	
King et al. (2004)	Prenatally malnourished (PMN) pups	Rats	Males	PN7, PN30	Reduced cell proliferation in the fascia dentata at PN7, but significantly higher on PN30.	
Oomen et al. (2009)	24 hMD on PN3	Rats	Males (M) females (F)	PN4, PN21	SEX DIFFERENCES found between control males and females. PN4: no changes in cell proliferation. PN21: (M) decreased cell proliferation, no effect on cell survival, increase in cell differentiation. (F) no effect on cell proliferation or cell survival, reduced cell differentiation.	
Racekov et al. (2009)	MS180 from PN1-PN14, or PN1 – PN21	Rats	Males	PN14, PN21, PN28	MS decreased proliferation in the Rostral Migratory Stream in all experimental groups.	
Oreland et al. (2010)	MS15 or MS360 from PN121	Rats	Males	PN22	MS360 decreased number of neurons and cell density in the DG, compared to MS15.	
Baek et al. (2011)	MS360, (PN1-14), and early weaning at PN15	Rat	Males	PN28	Decreased cell survival and cell differentiation. Reduced cell density in the DG.	
Lajud et al. (2012a)	MS180 from PN2 – 14	Rat	Males	PN15	MS180 decreased cell survival and cell differentiation. Reduced cell density in the DG.	Increased depressive – like behavior; no effect on anxiety.
Lajud et al. (2012b)	MS 180, PN2 – 14	Rats	Females	PN15	Decreased cell survival in the DG.	
Baek et al. (2012)	Early weaning on PN14 and later isolation	Rats	Males	PN35	Decreased cell proliferation in the DG.	
Wang and Gondr-Lewis (2013)	MS180 from PN2-21 with or without prenatal nicotine exposure	Rats	Males and females	PN14	MS180 increased pyramidal neurons in CA1. In the DG, MS180 decreased number of granule neurons.	
Lajud et al. (2013)	Prolactin, vehicle, or left undisturbed from PN1 – 14	Rats	Males, females	PN15	No differences in cell number or cell density between control males and females. Prolactin decreased cell survival in the DG.	
Naninck et al. (2014)	ES Limited nesting PN2 – 9	Mice	Males (M) females (F)	PN9, adulthood	PN9: increased cell proliferation and differentiation and number of immature cells in the DG. Adulthood: (M) reduced cell survival of adult born neurons. (F) No differences observed in adult females.	No effect on anxiety in the EPM, similar depressive- like behavior as controls.
Loi et al. (2014)	Comparison of prenatal, and postnatal stress (24 h MD)	Rats	Males (M) females (F)	Juveniles, adulthood	24 h MD enhances neurogenesis until the onset of puberty in male rats. Reduced neurogenesis in females prior puberty. The effect subsides in adulthood.	
Huot et al. (2002)	MS180 or MS15, on PN2 – 14	Rats	Males	Adulthood	Decreased mossy fiber density in the stratum oriens region but no change in volume of the DG.	
Mirescu et al. (2004)	MS 180, PN2 – 14	Rats	Males	Adulthood	Decreased cell proliferation and decreased immature neuron production in the DG.	
Fabricius et al. (2008)	24 h MD on PN9	Mice	Males and females together	Adulthood	Decreased cell number and cell proliferation in the DG in MD mice.	Reduced anxiety in the elevated plus maze.
Aisa et al. (2009)	MS180 PN2 – 21	Rats	Males	Adulthood	Reduced cell proliferation. Synaptic plasticity markers were decreased (NCAM, and synaptophisin, BDNF mRNAs).	
Karakaş et al. (2009)	24 h MD on PN4, PN9, or PN18	Rat	Males (M) females (F)	Adulthood	MD produced decreased the number of synapses in CA1 and CA3 subregions of the hippocampus. No differences between M and F were found.	
Kikusui and Mori (2009)	Early weaning in mice at PND15	Mice	Males (M) females (F)	Adulthood	M showed decreased BDNF levels in the hippocampus and prefrontal cortex, and reduced cell survival in theDG. No effect on F.	
Bagot et al. (2009)	Maternal care (high or low licking and grooming)	Rats	Males	Adulthood	Less dendritic complexity in low LG offspring compared to high LG.	
Oomen et al. (2010)	24 hMD on PN3	Rats	Males	Adulthood	MD decreased cell proliferation, cell survival, and neuronal differentiation. MD reduced total cell number in the caudal part of DG.	MD impaired spatial learning but enhanced contextual fear conditioning.
Leslie et al. (2011)	Unpredictable MS180 from PN1 – 14	Mice	Males and females together	Adulthood	No change in cell proliferation in the DG, but reduced cell survival and differentiation into neurons. Plus, less complex dendritic arborization and fewer dendritic spines.	
Oomen et al. (2011)	24 h MD on PN3	Rats	Females	Adulthood	No significant differences between groups in proliferation, cell survival or neuronal differentiation. MD reduced total cell number and cell density in the GCL.	No differences in spatial learning and contextual fear conditioning . MD improved amygdala-dependent fear memory.
Hulshof et al. (2011)	MS180 from PN1 – 14.	rats	males	adulthood	Cell proliferation was decreased in the DG of MS animals. Cell differentiation and survival were not altered.	No changes in anxiety-like behavior, explorative behavior and social interaction, but affected cognitive function (object recognition task).
Hays et al. (2012)	Morphine injections or exposure to MS 480	Mice	Males	Adulthood	Both neonatal stress or morphine treatment increased hippocampal neurogenesis in adult mice.	
Korosi et al. (2012)	Review		Males, females	Different ages		
Suri et al. (2013)	MS	Rats	Males	Adulthood	Enhanced hippocampal neurogenesis, with enhanced BDNF levels.	
Feng et al. (2014)	MS	Rats	Males	Adulthood	Increased cell proliferation in the subventricular zone, cortical layer 1 and hippocampal dentate gyrus.	

*We show a summary of the studies addressing the effects of early life adversity on different parameters of developmental neurogenesis, indicating sex, species, type of stress exposure, and age of the animals. Effects on neurogenesis in adult animals are also indicated, as well as long term effects on emotional behavior, where applicable.*

In adults, SGZ neurogenesis has been studied with divergent results. For instance, Mirescu et al. (2004) reported that male adult rats subjected to MS180 exhibited decreased cell proliferation and survival with inadequate responses to stress; while Hulshof et al. (2011) observed that cell proliferation in the DG was decreased in adult MS180 rats but not cell survival. Other studies found that adult MS mice had similar rates of proliferating cells in the DG as control groups, but presented a lower survival rate and differentiation (Leslie et al., 2011). Additionally, male adult mice that were early weaned showed a reduced number of BrdU+ cells in the DG (Kikusui and Mori, 2009). In contrast, several studies found an increase in cell proliferation in adult animals, previously subjected to MS180 (Suri et al., 2013; Feng et al., 2014). In a subset of experiments, adult MS mice (8 h/day) exhibited an enhanced hippocampal neurogenesis in adulthood (Hays et al., 2012). Very few studies are done in adult females. Adult MD females showed reduced granule cell number and density in the DG (Oomen et al., 2010, 2011) or presented no effect. Despite a reduced neurogenesis before puberty (Loi et al., 2014) females subjected to limited nesting showed no effect in adulthood (Naninck et al., 2014). These results suggest that protective factors could take place in the female brain.

In summary, most of the studies show a trend of a decreased proliferation and/or a decreased cell survival in the DG of male and female rodents immediately after stress exposure, which could affect mainly the tertiary dentate matrix neurogenic niche. In adulthood, direction of changes is variable. In males, initial changes are sometimes followed by an increase, or by a permanent decrease in these parameters. In females, it seems that early effects of stress on neurogenesis subside in adulthood. In spite of the variety and direction of the changes that take place in the DG, it is assumed that early life stress induces such alterations to enable the individual to cope with future adversity in life (Bagot et al., 2009).

## LONG TERM CONSEQUENCES IN EMOTIONALITY

Early adversity has been linked to the development of psychiatric illness. A neurogenic hypothesis of depression was formulated, after findings of reduced hippocampal volumes in depressed patients, and the fact that chronic stress decreases hippocampal neurogenesis, and increases the risk to develop depression (Jun et al., 2012). Further, antidepressants were found to enhance hippocampal neurogenesis (Santarelli et al., 2003). However, the correlation of long term behavioral changes with hippocampal neurogenesis changes is still controversial due to several observations that neurogenesis and emotionality are independently regulated (Petrik et al., 2012). Proposals to reconcile the different results have been adressed (Eisch and Petrik, 2012).

Practically all the reports in animal models of long term consequences of early adversity in emotionality use adults. Studies vary, from no effects, to increases in anxiety and/or in depressive – like behavior in males (Newport et al., 2002; Daniels et al., 2004; Lee et al., 2007; Lajud et al., 2012a; Girardi et al., 2014; Nishi et al., 2014). In females, results are scarce but point to a lack of effect on contextual fear conditioning (Oomen et al., 2011), or anxiety (screened in the elevated plus maze; Grissom et al., 2012), but increase social anxiety (Tsuda and Ogawa, 2012). Fewer studies report changes in neurogenesis (**Table 1**) together with effects on emotionality (Fabricius et al., 2008; Hulshof et al., 2011; Oomen et al., 2011; Lajud et al., 2012a; Naninck et al., 2014). Thus, it seems that in rodents, males are more vulnerable to the effects of early adversity than females.

## EARLY LIFE STRESS EFFECTS ON DG DEVELOPMENT AND SOME POSSIBLE MEDIATORS

We highlight two well-known factors mediating the stress response, and two peptide messengers as potential mediators.

## GLUCOCORTICOIDS

During the first 2 weeks of life, rat pups experience a stress hyporesponsive period (SHRP) due to a markedly reduced adrenocortical response to stress (Sapolsky and Meaney, 1986). GC administration during the first postnatal week decreases granule cell survival, and results in a significant increase in the density of both cell proliferation and death, within the hilus neurogenic niche (Gould et al., 1991). Since then, increased circulating GC levels have been proposed as the main mediators of early life stress effects on hippocampus developmental neurogenesis (Gould and Tanapat, 1999). GCR gene expression is present within the developing brain since early fetal development (Yi and Baram, 1994), and is maximally expressed in the DG between PND10 and 16 (Yi and Baram, 1994); however, other mediators could be involved in the psychopathology of early life stress (Schmidt et al., 2009; Lajud et al., 2012a; Horii-Hayashi et al., 2013; Liao et al., 2014). GCR regulate CRH gene promoter in the mature brain. In the neonate, second messenger cascades are not yet functional, and GC levels fail to modify CRH expression. Thus, GCR may mediate different functions in the developing neurons, possibly mediated by trophic effects (Yi and Baram, 1994).

## CORTICOTROPIN RELEASING HORMONE

The CRH is considered the link between early life adversity and adult vulnerability (Brunson et al., 2003). CES and MS permanently increase CRH expression within the hippocampus (Ivy et al., 2010; Wang et al., 2014). Administration of CRH into the brains of infant rats recapitulates some of the long term effects associated with early life stress, even when GC levels are clamped at physiological levels (Brunson et al., 2001; Wang et al., 2012). Blockade of CRHR1 signaling during adulthood significantly attenuated the hippocampal synaptic dysfunction, and memory defects in maternally separated rats (Wang et al., 2014), and treatment blocking CRHR1 from PN10-17 prevented ELS – induced augmentation of hippocampal in middle-aged rats (Ivy et al., 2010). Notably CRHR1, but not GCR, antagonism during the developmental critical period attenuated ES – induced endocrine alterations (Ivy et al., 2010; Liao et al., 2014). Moreover, a specific population of Cajal-Retzius-like CRH-expressing neurons was characterized during early postnatal hippocampus and these cells seem to contribute to the establishment of hippocampal connectivity (Chen et al., 2001).

## PROLACTIN

Prolactin (PRL) is a pleiotropic hormone promoting a vast array of effects (Freeman et al., 2000). PRL is released in response to stress, regulates hippocampal and SVZ neurogenesis, and modulates anxiety and HPA axis reactivity (Torner et al., 2001, 2009; Shingo et al., 2003). PRL enters the brain through its receptors, located in the choroid plexus cells (Walsh et al., 1987). Daily PRL administration (PN1 to PN14) induced a decrease of DG neurogenesis of PN15 pups (Lajud et al., 2013), and increased depressive – like behavior, in adult male and female rats (Lajud et al., 2013). Studies showed that PRL enhances CRH (Blume et al., 2009) and AVP expression (Donner and Neumann, 2009; Vega et al., 2010). Additionally PRL is cleaved to produce vasoinhibin, which has opposite actions of the native hormone (Zamorano et al., 2014). Thus, PRL could contribute to stress programming.

## CITOKINES

Prenatal maternal infections increase the risk of developing schizophrenia or autism in the offspring (Kofman, 2002; Musaelyan et al., 2014). Immune activation during the perinatal period increases cytokine production, particularly of Interleukin 1 beta (IL-1b) and tumor necrosis factor-α, in the hippocampus (Diz-Chaves et al., 2012). Treatment of hippocampal neurospheres with IL-1b showed an antiproliferative, antineurogenic and pro-gliogenic effects (Green et al., 2012). Further, IL-1b reduced the serotonergic differentiation of cultured neurospheres in a dose-dependent manner (Zhang et al., 2013). Several studies reported either increases or decreases in cell proliferation or cell survival in the hippocampus offspring depending on the time of exposure and the time of neurogenesis assessment (Musaelyan et al., 2014). Further, mice pups given an immune challenge at PN9, showed reduced cell proliferation, and reduced cell number of neural progenitors at PN41 (Järlestedt et al., 2013). Thus, cytokines play an important role during brain development.

## CONCLUDING REMARKS

Early adversity disrupts the normal concentrations of important neurotransmitters, peptides, hormones, cytokines or their receptors, which are either expressed in the brain or enter the brain compartment during development. These alterations influence the local milieu in the DG, possibly affecting the tertiary dentate matrix and producing a decrease of granular neurons, a decreased cell survival and/or differentiation (**Figure 1**). Compensatory mechanisms, such as differential expression of neurotrophic factors, might, or not, induce a secondary increase of granular neuron synthesis at adult ages, in both male and female rodents. In any case, the alteration of the development of the structure and compensatory mechanisms induce permanent changes in hippocampal function, which are sometimes accompanied by increased anxiety or depressive-like behavior in males.

## AUTHOR CONTRIBUTIONS

Author Naima Lajud, managed the literature searches, made substantial contributions to the conception or design of the work; wrote a part of the manuscript and revised the work critically, and designed **Figure 1**. Author Luz Torner made the conception and design of the work, managed the literature searches, made **Table 1**, wrote a part of the manuscript, revised the work critically, and wrote the final draft. Both authors contributed to and have approved the final manuscript.

## Conflict of Interest Statement

The authors declare that the research was conducted in the absence of any commercial or financial relationships that could be construed as a potential conflict of interest.
